# Fluctuations in barriers to medication treatment for opioid use disorder prescribing over the course of a one-year external facilitation intervention

**DOI:** 10.1186/s13722-021-00259-1

**Published:** 2021-08-06

**Authors:** Allison M. Gustavson, Marie E. Kenny, Jennifer P. Wisdom, Hope A. Salameh, Princess E. Ackland, Adam J. Gordon, Hildi J. Hagedorn

**Affiliations:** 1 Veterans Affairs Health Services Research and Development Center for Care Delivery and Outcomes Research, Minneapolis Veterans Affairs Health Care System, 1 Veterans Drive, Mail Code #152, Minneapolis, MN 55417 USA; 2 Wisdom Consulting, New York, 1133 Broadway #1205, New York, NY 10010 USA; 3 Department of Medicine, University of Minnesota, Minneapolis, USA; 4 Vulnerable Veteran Innovative PACT (VIP) Initiative; Informatics, Decision-Enhancement, and Analytic Sciences Center (IDEAS), VA Salt Lake City Health Care System, 500 Foothill Drive, Salt Lake City, UT 84148 USA; 5 Program for Addiction Research, Clinical Care, Knowledge and Advocacy (PARCKA), University of Utah School of Medicine, Department of Internal Medicine, Division of Epidemiology, Salt Lake City, UT USA; 6 Department of Psychiatry, University of Minnesota School of Medicine, Minneapolis, MN 55455 USA

**Keywords:** Implementation, Facilitation, Medication treatment, Opioid use disorder, Qualitative

## Abstract

**Background:**

The Veterans Health Administration (VHA) is invested in expanding access to medication treatment for opioid use disorder (MOUD) to save lives. Access varies across VHA facilities and, thus, requires implementation strategies to promote system-wide adoption of MOUD. We conducted a 12-month study employing external facilitation that targeted MOUD treatment among low-adopting VHA facilities. In this study, we sought to evaluate the patterns of perceived barriers over 1 year of external implementation facilitation using the integrated Promoting Action on Research Implementation in Health Services (i-PARIHS) framework.

**Methods:**

We randomly selected eight VHA facilities from the bottom quartile of the proportion of Veterans with an OUD diagnosis receiving MOUD (< 21%). The 1-year external implementation intervention included developmental evaluation to tailor the facilitation, an on-site visit, and monthly facilitation calls. Facilitators recorded detailed notes for each call on a structured template. Qualitative data was analyzed by coding and mapping barriers to the constructs in the i-PARIHS framework (*Innovation*, *Recipients*, *Context*). We identified emerging themes within each construct by month.

**Results:**

Barriers related to the *Innovation,* such as provider perception of the need for MOUD in their setting, were minimal throughout the 12-month study. Barriers related to *Recipients* were predominant and fluctuated over time. *Recipient* barriers were common during the initial months when providers did not have the training and waivers necessary to prescribe MOUD. Once additional providers (*Recipients*) were trained and waivered to prescribe MOUD, *Recipient* barriers dropped and then resurfaced as the facilities worked to expand MOUD prescribing to other clinics. *Context* barriers, such as restrictions on which clinics could prescribe MOUD and fragmented communication across clinics regarding the management of patients receiving MOUD, emerged more prominently in the middle of the study.

**Conclusions:**

VHA facilities participating in 12-month external facilitation interventions experienced fluctuations in barriers to MOUD prescribing with contextual barriers emerging after a facilitated reduction in recipient- level barriers. Adoption of MOUD prescribing in low-adopting VHA facilities requires continual reassessment, monitoring, and readjustment of implementation strategies over time to meet challenges. Although i-PARIHS was useful in categorizing most barriers, the lack of conceptual clarity was a concern for some constructs.

**Supplementary Information:**

The online version contains supplementary material available at 10.1186/s13722-021-00259-1.

## Background

The rising prevalence of opioid use disorder (OUD) among Veterans [1] is of significant concern to the Veterans Healthcare Administration (VHA) given that provision of timely and evidence-based treatment saves lives and has implications for healthcare utilization [2–4], risk of human immunodeficiency virus (HIV) and hepatitis C (HCV) infection [5], societal costs [6], and quality of life [7]. Medication treatement for opioid use disorder (MOUD, including buprenorphine, methadone, and naltrexone) is the evidence-based, first-line treatment for OUD [8]. The VHA is invested in increasing access to MOUD [1]. Access varies widely across VHA facilities, with prescribing rates of MOUD for patients with OUD diagnoses ranging from 1 to 68% depending on facility [1, 9, 10]. Thus, system- wide adoption of MOUD requires implementation strategies, such as external implementation facilitation, to promote rapid and sustainable change in healthcare delivery. To address this gap in Veteran care, we conducted a 12-month study employing external implementation facilitation that targeted MOUD treatment among low-adopting VHA facilities [11]. Low-adopting facilities were categorized as those in the bottom quarter of the proportion of Veterans receiving MOUD over the number of Veterans with OUD diagnoses. Although the initial target was to increase MOUD use in the substance use disorder specialty clinics, the external implementation facilitation intervention expanded to promote prescribing of MOUD in other clinics-- including primary care, mental health, and pain specialty clinics-- depending on local facility needs and interests. The addition of these clinics into the implementation intervention offered an opportunity for greater impact on MOUD prescribing rates as issues with opioid use are frequently first identified in these settings and many Veterans are reluctant to follow through with a referral to substance use disorder specialty care due to stigma related to substance use disorders [12]. When a Veteran is seen and managed at a single clinic with a single provider or team, the potential for fragmented care and the associated negative outcomes is substantially reduced [13].

The external implementation facilitation strategy employed to enhance adoption of MOUD in low-adopting VHA facilities was guided by the integrated-Promoting Action on Research Implementation in Health Services (i-PARIHS) framework, which hypothesizes that implementation success of a clinical innovation is driven by active facilitation with recipients (e.g., providers, leaders, patients) within their context (e.g., clinical setting) [14]. Our approach to external implementation facilitation was multi-faceted and included: developmental evaluation via key stakeholder interviews to tailor the facilitation,an on-site visit with active goal setting, education, and MOUD training. and monthly facilitation calls [11]. During the monthly facilitation calls over 1 year, local VHA facility teams met with the external implementation facilitation team to discuss progress on action plans that were collaboratively developed at the site visit. The facilitators were able to connect the local implementation teams with subject matter expert consultations and provide access to resources such as training links, manuals, clinical note templates, and standard operating procedures.

Barriers to adoption of evidence-based practiced inherently exist; yet, several implementation strategies such as facilitation have a strong-evidence base for overcoming these barriers. However, significant gaps remain in our understanding of whether barriers vary across time, how to measure that variability during an implementation study, and how to adjust implementation strategies at appropriate times to maximize implementation success. We sought to 1) identify barriers to MOUD provision, as described qualitatively by key stakeholders, change over time in response to the 12-month external facilitation intervention and 2) evaluate the usefulness of i-PARIHS as an organizing framework for identifying and addressing barriers to implementation.

## Methods

### Sample

The protocol for the Advancing Pharmacological Treatments for Opioid Use Disorder (ADaPT-OUD) study is described elsewhere [11]. Briefly, we provided an external facilitation implementation intervention to eight VHA facilities randomly selected out of the 35 VHA facilities considered to have low provision of MOUD based on their presence in the lowest quartile of proportion of Veterans receiving MOUD over the number of Veterans with OUD diagnoses. The external facilitation implementation intervention consisted of a site-specific report developed from rapid qualitative analysis of interviews with site stakeholders (e.g., facility and clinic-level leadership, primary care providers, mental health providers, substance use disorder providers) conducted prior to the site visit. The report included the site’s strengths, challenges, and needed resources.

Following development of the site report, the primary external facilitators (HJH and AJG) visited each site for 1–2 days and provided a review of the site’s report, education for clinicians (e.g., providers from substance use disorder, mental health, primary care, or pain clinics) on MOUD topic areas and available resources, the first four hours of the training for providers to obtain their Drug Enforcement Administration (DEA) X-waiver required for buprenorphine prescribing, and engagement of a local implementation team in identifying goals and strategies to achieve goals in the form of an action plan. By the end of the site visit, each site had identified goals and the members of the local implementation team. Implementation team members varied by site but included key stakeholders who represented a diverse array of disciplines (e.g., social work, pharmacy, psychiatry, medical providers, nursing) across a number of clinics in the VHA facility (e.g., substance use disorder, primary care, mental health). The site visits were followed by 1 year of monthly telephone meetings with the external facilitation team to track progress, document new or persisting implementation barriers, and identify solutions to challenges.

### Data collection and analysis

Researchers took extensive notes during all telephone meetings using a structured template (see Additional file 1) outlining the site’s action plan and any barriers the facility encountered while enacting the action plan. Meetings were conducted by reviewing progress on each goal, following-up on action items identified in the previous call, and problem-solving any challenges. After the meeting, the notes were distributed to the entire team to review for accuracy and provide additional comments that were then updated in the notes. This qualitative study was not planned *a priori*; however, as part of the facilitation intervention, detailed structured notes were taken by two study team members (MEK and HAS) during every facilitation call to allow the facilitator to track barriers across calls. As this information was useful to the facilitator during the intervention, we felt that a more formal qualitative analysis of the notes would provide interesting and useful information regarding the implementation barriers experienced over the course of the intervention.

We used the notes collected from each site during the monthly meetings (1-year duration) as qualitative data for analysis. First, two coders (MEK and HAS) independently reviewed and deductively coded notes from monthly call meetings (12 months per site) from each of the eight sites. The coding scheme used the integrated Promoting Action on Research Implementation in Health Services (i-PARIHS) framework, consisting of *Innovation*, *Context*, and *Recipients* constructs [14]. We operationalized *Innovation* as the clinical intervention characteristics (i.e., MOUD), including information derived from the evidence -base, clinical experience and practice-based knowledge, patient experiences and values, and local or organizational experiences and values. *Recipients* were operationalized as the characteristics of the people who enact and influence the MOUD implementation, such as their motivation, values and beliefs, goals, skills and knowledge, time, resources and support, and collaboration or teamwork. *Context* was operationalized by multiple layers (i.e., local, organizational, external health system) that can enact or constrain implementation. For each goal on the action plan, barriers were coded independently as a barrier for that goal, which allowed for the same barrier to be counted more than once if it occurred in another goal (see Additional file 1). After coding independently, the two coders (MEK and HAS) met to discuss discrepancies and come to a consensus. One researcher (JPW) reviewed the final coding and added sub-codes reflective of the sub-constructs under *Innovation*, *Recipients*, and *Context*. The entire research team then met four times to discuss any discrepancies and reach a consensus on the coding. Next, we calculated the frequency of each code (*Innovation*, *Recipients*, *or Context*) by month for all the sites together to create a visual representation of the data. The research team (AMG, MEK, HAS, PEA, AJG, HJH, JPW) then met to identify emerging themes in the qualitative data based on the coding and code frequencies by month. This iterative process of analysis for emergent themes continued until consensus was reached.

## Results

### Patterns of *innovation* barriers over 1-year of external facilitation

**Fig. 1:**
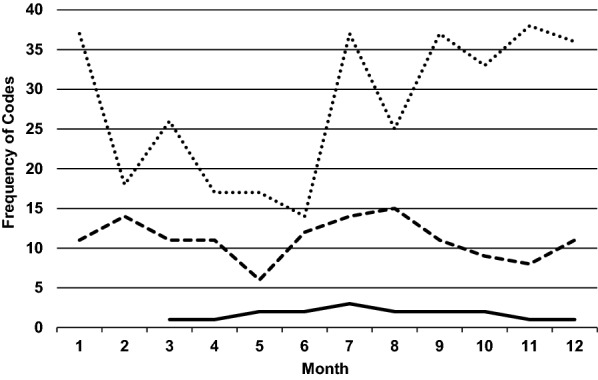
Frequency of codes for i-PARIHS barriers during monthly calls over 1 year. The solid line indicates *Innovation* barriers, the dotted line *Recipient* barriers, and the dashed line *Context* barriers

Figure 1 depicts the barriers across all eight sites over the 12-month period as measured by the number of times each barrier was reported on the monthly facilitation calls. Barriers related to the construct of *Innovation* (i.e., MOUD) were minimally referred to during any facilitation calls. Barriers related to the *Innovation* were rare throughout the 12- month external facilitation intervention suggesting that key stakeholders agreed with the evidence supporting the clinical intervention (i.e., MOUD provision). The barriers coded as *Innovation* tended to focus on the appropriateness of MOUD for patients who were taking prescribed opioids for chronic pain. Providers had questions about when such patients met criteria for the diagnosis of OUD and were appropriate to receive MOUD. Providers also raised questions about where (e.g., primary care, mental health pain specialty clinics, or in substance use disorder specialty clinics) these patients would receive initiation of MOUD and then long-term management of the treatment. When a facility’s action plan included efforts to expand MOUD prescribing into other clinics outside of the substance use disorder clinic, the facilitation team would assist with providing targeted education on OUD diagnosis, resources and education regarding the diagnosis of OUD, and resources regarding criteria for use of different MOUD.

### Patterns of *recipient* barriers over 1-year of external facilitation

The pattern of *Recipient* barriers fluctuated over the 1-year external facilitation intervention. *Recipient* barriers were reported more frequently than the *Context* and *Innovation* constructs during all monthly calls. We observed fluctuations in *Recipient* barriers related to provider skills and knowledge throughout the 12-month external facilitation intervention with such barriers starting out high (month 1), declining following initial training/education efforts (months 2–6), and then increasing again (month 7) as facilities expanded efforts to new clinics or dealt with chronic staff turnover. Initial facilitation efforts at the site visit and early implementation focused on providing education to fill gaps in knowledge and skills, which may explain the drop in *Recipient* barriers. However, the later rebound of these barriers reinforces the need for ongoing education and training to promote both expansion to new clinics and sustainability in the face of staff turnover. Throughout the study, barriers related the lack of resources and support for staff were also common and related to resistance from facility leadership to restructure existing resources (e.g., staffing, clinical caseloads). When this was the case, the facilitation team assisted the local clinics/staff with arranging meetings with facility leadership to discuss resource allocation or developing proposals designed to lay out specific resources needed to prescribe MOUD in non-substance use related clinics such as primary care, pain specialty clinics, and mental health. These proposals were then elevated to facility leadership for approval.

### Patterns of *context* barriers over 1-year of external facilitation

As shown in Fig. 1, we observed a steady pattern of *Context* barriers over the first 5 months. During the next three months (months 6–8) we observed a slight, steady increase in the number of *Context* barriers referenced per facilitation call. The remaining calls (months 9–11) showed a downward trend in the number of *Context* barriers mentioned per monthly call across all facilities. Qualitative data suggested *Context* barriers were largely related to organizational and clinic-level infrastructure. These issues included who (and in what clinic) could provide MOUD, the frequency of follow-up care after MOUD initiation, and how medications would be ordered and stocked for in-clinic initiation. Reports of these barriers were generally consistent over time with a small increase just prior to the mid-point of the 12-month facilitation duration. The qualitative data, along with the experience of the facilitation team, suggests that the facilities generally started out with the belief that education and training (*Recipients*) would resolve their implementation barriers and lead to change. Therefore, most of the time and energy at the start of the implementation phase was focused on training and education. Once these issues resolved, facilities moved on to expand prescribing to other clinics, where they began to encounter contextual issues. The facilitation team assisted with these issues by providing resources for and examples of clinic standard operating procedures, clinical note templates for documentation, scheduling grids, and options for getting medications to patients before the initiation appointment. In addition, the facilitation team used their network of experienced providers at other facilities to set- up consultations regarding how clinics functioned in other facilities. This cross-facility consultation allowed struggling sites to hear about other successful facilities’ experiences and responses to barriers and then translate them to their own facilities.

## Discussion

This study indicates barriers to adoption of MOUD in low-adopting VHA facilities fluctuated over a 1-year external implementation facilitation intervention with variations in barriers emerging during monthly facilitation calls. Barriers related to the *Innovation* itself were minimal throughout the 12-month study with barriers related to *Recipients* being the most prominent and subject to fluctuation in response to the external facilitation intervention. *Context* barriers emerged more prominently in the middle of the study yet remained steady and low compared to the number of references to *Recipient* barriers.

The nature of the barriers identified were similar to other studies exploring adoption of MOUD including scarce availability of prescribers, limited staff training, inadequate space, and lack of patient and provider interest. [1, 15, 16] Our study adds to this literature by exploring temporal trends in barriers interpreted through the i-PARHIS lens, that were specifically targeted and addressed as a result of an external facilitation implementation intervention.

The MOUD clinical intervention (*Innovation*) did not seem to be a barrier throughout the implementation study suggesting general agreement with MOUD as first line treatment for OUD, while barriers under *Context* and *Recipients* were prominent and fluctuated across time. This suggests although low-adopting VHA facilities agreed with the evidence for and the objective of increasing access to MOUD, they were constrained in their efforts by complex barriers related to *Context* and *Recipients*. Providers (*Recipients*) were required to invest considerable time and effort to obtain the necessary training to prescribe MOUD while also facing contextual hurdles such as credentialing procedures and state restrictions of who can prescribe (e.g., nurse practitioners are not allowed to independently prescribe buprenorphine for OUD in some states).

The fluctuation of *Recipients* and *Context* barriers in response to the external facilitation methods suggests barriers emerged at different time points and, thus, required multiple problem-solving discussions over the course of the monthly calls. For example, the first few months of the external facilitation addressed immediate needs to increase MOUD provision by providing education and training opportunities for providers at the site to obtain their waiver to prescribe MOUD, which appears to correspond to a drop in *Recipient* related barriers. However, once providers were waivered and credentialed, *Context* barriers emerged more prominently, confirming education and training alone do not lead to implementation of complex changes to clinical practice. Teams encountered multiple difficult and complex barriers related to clinic organization, facility policies, and the lack of cross-clinic coordination. *Recipient* barriers reemerged as teams attempted to expand access to additional clinics and the *Recipients* (staff) changed over the course of the 12-month study due to organizational restructuring, new staff being hired, and staff leaving the VA or that specific facility’s employment.

Although i-PARIHS is designed to represent the dynamic interaction between the factors that influence implementation, we found the definitions of the sub-constructs lacked precision and gave way to potential overlap across sub-constructs. For example, inadequate time for staff to complete necessary training or burdensome credentialling processes to provide MOUD could be coded under *Context,* sub-category *Organizational Structures and* *Systems* because the system dictates the policies for staff training and credentialling. However, we coded these barriers under *Recipients,* sub-category *Time, resources, and support* since the inability to get providers the necessary training and approval for MOUD provision was considered essential to adoption of MOUD. We initially selected the i-PARIHS framework in the pre-planning phase as in this conceptual framework successful implementation relies on facilitation (i.e., our implementation intervention) that aligns the *Innovation* (i.e., MOUD) with the *Recipients* within the constraints of their *Context* [14]. This led to using a team-based consensus on the coding, which may lack reliability across different research teams. Since the expansion of sub-constructs in the transition to the i-PARIHS framework (from the original PARIHS), the framework now aligns much more closely with the Consolidated Framework for Implementation Research (CFIR) [17]. However, some barriers would have fallen into different constructs under CFIR. For example, i-PARIHS categorizes a barrier of “Time, Resources, and Support,” under *Recipients*, whereas the CFIR places these barriers under the *Inner Setting* as contextual factors that influence implementation. This highlights the ongoing challenge in implementation science of developing a common language for implementation determinants with a consistent understanding of their definitions [18].

This study has several limitations. First, we cannot extrapolate the pattern of i-PARIHS barriers observed here to other VHA facilities with higher adoption of MOUD, outside of the VHA system, or to implementation of other evidence-based clinical interventions. We also acknowledge that qualitative results are not generalizable but may be transferrable. However, the general principles may still apply in that initial focus of facilitation may be at the recipient level (e.g., education) to generate short term wins, followed by the emergence of contextual factors that require facilitating infrastructure and policy change at the facility or organizational level. Secondly, although we expected to use the i-PARIHS framework to guide and analyze our implementation efforts, the monthly facilitation records did not solicit an exhaustive list of barriers and, thus, barriers were only recorded if the participants on the call mentioned them. In addition, clinical duties often prevented some implementation team members from attending calls, so their perspectives were not captured; however, we emailed the summaries of call notes and offered all implementation team members the opportunity to contact us with additional updates, questions, or concerns if they missed the call. Finally, the quantification of qualitative results is subject to bias given the many decisions made by investigators during the process. However, while we acknowledge that the frequencies may not be exact or easily reproducible, the quantification allowed us to observe the prevalence and timing of themes throughout the external facilitation intervention.

This study’s strength is that it provides insight in the fluctuation of barriers across an implementation intervention to prompt implementation scientists to consider measuring and analyzing these patterns to inform the optimal timing and type of implementation strategies. Future work will explore the effects of timing, type, and frequency of facilitation strategies employed on the implementation outcome through implementation studies.

## Conclusions

Enhancing the adoption of MOUD provision through targeted implementation interventions, such as external facilitation, is critical to reducing variability in practice and enhancing access to evidence-based care. Barriers to MOUD provision for low adopting VHA facilities fluctuated across the 12-month external facilitation intervention with contextual barriers emerging after a facilitated reduction in recipient- level barriers occurred. Recipient barriers reemerged as actionable goals expanded to new settings or staff/leadership turn-over occurred. Implementation of MOUD care is complex and requires continual reassessment, monitoring, and readjustment to meet anticipated and unanticipated challenges. Measuring and monitoring patterns of barriers and responses to implementation strategies is needed to correctly time and tailor strategies for optimal impact on clinical practice. We also found it challenging to apply the i-PARIHS implementation framework to reliably categorize barriers to understand changes across time, which can impact precision in matching barriers to external facilitation implementation strategies**.** Ongoing research toward conceptual clarity of key implementation constructs across implementation frameworks will advance the practice of implementation science.

## Supplementary Information

**Additional file 1: Table S1.** Structured template for notes during monthly site facilitation calls.

## Data Availability

Deidentified data can be obtained by contacting the corresponding author.

## Abbreviations

VHA: Veterans’ Health Administration
MOUD: Medication for opioid use disorder
OUD: Opioid use disorder
i-PARIHS: integrated Promoting Action on Research Implementation in Health Services frameworks
ADaPT-OUD: Advancing Pharmacological Treatments for Opioid Use Disorder study
DEA: Drug Enforcement Administration
CFIR: Consolidated Framework for Implementation Research
