# Supplementary material for: Fluctuations in barriers to medication treatment for opioid use disorder prescribing over the course of a one-year external facilitation intervention
Source: Addict Sci Clin Pract. 2021 Aug 6;16:51. doi: 10.1186/s13722-021-00259-1 (PMC8343892; doi:10.1186/s13722-021-00259-1)
Supplement: Supplementary file 1 — Additional file 1: Table S1. Structured template for notes during monthly site facilitation calls. [file 13722_2021_259_MOESM1_ESM.docx]

| Facilitator(s): | Site: | | Date: |
| --- | --- | --- | --- |
| Attendees on call (list): | |  | |
| Action plan Goal #1: | | | |
| Progress made: | | | |
|  | | | |
| Barriers encountered: | | **Strategies to employ:** | |
|  | |  | |
| Steps to be taken before next call: | **Assigned to:** | | **Target completion date:** |
|  |  | |  |
|  |  | |  |
| Action plan Goal #2: | | | |
| Progress made: | | | |
|  | | | |
| Barriers encountered: | | **Strategies to employ:** | |
|  | |  | |
| Steps to be taken before next call: | **Assigned to:** | | **Target completion date:** |
|  |  | |  |
|  |  | |  |
| Action plan Goal #3: | | | |
| Progress made: | | | |
|  | | | |
| Barriers encountered: | | **Strategies to employ:** | |
|  | |  | |
| Steps to be taken before next call: | **Assigned to:** | | **Target completion date:** |
|  |  | |  |
| Action plan Goal #4: | | | |
| Progress made: | | | |
|  | | | |
| Barriers encountered: | | **Strategies to employ:** | |
|  | |  | |
| Steps to be taken before next call: | **Assigned to:** | | **Target completion date:** |
|  |  | |  |

**Additional file 1 Table S1.** Structured template for notes during monthly site facilitation calls.
